# The Role of Rheumatologist in Smoking Cessation of Ankylosing Spondylitis Patients: A Single-Center Cross-Sectional Study

**DOI:** 10.7759/cureus.45461

**Published:** 2023-09-18

**Authors:** Mehmet Nur Kaya, Seda Yurumez, Emre Tekgöz, Özlem Kılıç, Muhammet Çınar, Sedat Yılmaz

**Affiliations:** 1 Rheumatology, Gülhane Training and Research Hospital, Ankara, TUR

**Keywords:** smoking, herbal supplement, smoking cessation, rheumatologist, ankylosing spondylitis

## Abstract

Objectives: Smoking has a well-established detrimental effect on the prognosis and treatment success in patients with ankylosing spondylitis. It is important to encourage and convince patients to quit smoking. We aimed to evaluate the contribution of rheumatologists to smoking cessation in patients with ankylosing spondylitis.

Methods: This single-center cross-sectional study was conducted in a tertiary research hospital between March 2022 and June 2022. The data related to demographics, smoking status, duration of smoking, average number of cigarettes smoked per day, reasons for quitting smoking, and methods of quitting smoking were obtained through face-to-face interviews.

Results: This study was carried out on 308 patients. A total of 102 ankylosing spondylitis patients quit smoking. Of the patients who quit smoking, 39 (38.3%) patients quit smoking with a recommendation of a rheumatologist and 29 (28.4%) patients quit because of their concerns related to ankylosing spondylitis disease. The most commonly used methods for quitting smoking were herbal supplements in 40 (39.2%) patients and medication for smoking cessation in 40 (39.2%) patients.

Conclusions: It has been shown that about one-fifth of ankylosing spondylitis patients are not questioned by a rheumatologist about smoking. On the other hand, it was seen that the factor with the greatest effect on those who quit smoking was the rheumatologist. Therefore, rheumatologists should question all ankylosing spondylitis patients about smoking and encourage smokers to quit in order to achieve better outcomes in the long term.

## Introduction

Smoking is a serious public health concern and contributes to many chronic diseases, including various cancers and cardiovascular disorders. For the past two decades, smoking has been linked to a worsening of the progression of various rheumatic diseases. These effects are not limited to disease etiopathogenesis but may also interfere with disease progression. Smoking has been associated with both poor prognosis and treatment nonresponsiveness [1].

Ankylosing spondylitis (AS) is a chronic systemic inflammatory disease and the prototype of the spondyloarthritis family, which typically affects the sacroiliac joint, spine, peripheral joints, and entheses [2]. It is estimated that one in every 200 people is affected by AS, which becomes a significant health and socioeconomic problem [3]. Although the exact etiology of AS has not been established yet, along with others, cigarette smoking has also played a role in the onset and development of the disease [4]. Besides, a significant negative correlation has been shown between pain, fatigue, disease activity, functional status, physical mobility, and disease-related cumulative smoking in male patients with AS. From a different perspective, smoking is associated with more functional limitations, as evidenced by the finding that increased levels of smoking addiction were linked to an increase in Bath Ankylosing Spondylitis Metrology Index (BASMI) scores [5]. A cross-sectional study in an axial spondyloarthritis (axSpA) cohort showed that smoking was associated with increased disease activity and functional impairment. In axSpA participants reporting a continuous smoking history, increased cumulative smoking exposure was observed to have a significant association with the Bath Ankylosing Spondylitis Disease Activity Index (BASDAI) [4].

Smokers have a life expectancy that is at least 10 years shorter than that of non-smokers, and two-thirds of smokers die from a smoking-related disease. Although quitting smoking is very important, 90%-95% of smokers who try to quit start smoking again within a year [6]. Studies show that doctors' smoking cessation advice is a powerful motivator to promote smoking cessation. One study shows that physicians' advice on smoking cessation can increase the smoking cessation rate by 3% to 6% [7,8]. In another study, 68.8% of current smokers quit smoking with the advice of a doctor [8]. To the best of our knowledge, there is no study showing the effect of rheumatologist advice on smoking cessation rates in AS patients. In this study, we aimed to evaluate rheumatologists' questions about smoking habits and their effect on quitting smoking in patients with AS.

## Materials and methods

Study design and approval

This descriptive, single-center, and cross-sectional study was conducted at Gülhane Training and Research Hospital between January 2022 and March 2022. It was designed according to STROBE rules in article writing. This study was approved by the Ethics Committee of Gülhane Training and Research Hospital (Date: December 15, 2021 and Decision number: 2021/101) and all participants provided written informed consent. All procedures were carried out in accordance with the ethical rules and the principles of the Declaration of Helsinki.

Participants

This study included 308 AS patients who were followed up in the rheumatology outpatient clinic of Gülhane Training and Research Hospital. Inclusion criteria included participants who provided informed consent, were over 18 years of age, and met the diagnostic criteria for AS according to the modified New York criteria. Exclusion criteria included current pregnancy, an unstable systemic medical condition, those who did not have information about their smoking history, and those who did not provide an informed consent form.

Data collection and protocol

The previously prepared data collection form was filled in by two rheumatologists by interviewing each participant face-to-face. The data collection form consists of 10 questions about smoking status and demographic data. Demographic data include age, sex, body mass index (BMI), comorbidity, education, marital status, disease duration, disease onset age, alcohol use, drug therapy, and HLA-B27. Diabetes mellitus, hypertension, coronary artery disease, chronic kidney disease, and chronic obstructive pulmonary disease were evaluated as comorbid diseases.

The data on smoking were obtained from the questions asked to the participants about their smoking status, duration of smoking, average number of cigarettes smoked per day, whether doctors advised them to quit smoking, reasons for quitting smoking, and methods of quitting smoking.

Cigarettes are counted in terms of pack years. One pack-year was considered to be 20 cigarettes per day for one year of cigarette consumption. Smokers were divided into three categories: current smokers, former smokers, and non-smokers. In order to be considered “quit smoking,” the patients' statement that they had not smoked for at least six months was taken as a basis.

It was defined as quitting smoking with “the advice of a rheumatologist” when the physician stated that smoking would cause the progression of the disease and decrease the effectiveness of the treatment while patients with AS were under treatment. Patients with AS were defined as quitting smoking due to their concerns about their disease, without the advice of a rheumatologist, as the “cause of AS disease.” Patients with AS were defined as “voluntarily quitting” smoking due to the negative effects of smoking, without the presence of their current disease and the advice of a rheumatologist.

Study endpoints

The primary endpoint was the rheumatologist's questioning of smoking status and rheumatologist-recommended smoking cessation rate in AS patients. Secondary endpoints were smoking status and factors leading to smoking cessation in AS patients.

Statistical analysis

The sample size calculated based on previous work was calculated to yield an error alpha of 0.05 for a power of 0.95. For statistical analyses, IBM SPSS Statistics for Windows, version 26.0 (IBM Corp., Armonk, NY, USA) was used. The variables were investigated using visual (histograms, probability plots) and analytical methods (Kolmogorov-Smirnov) to determine whether they were normally distributed. Descriptive statistics were presented as mean ± standard deviation and median (minimum-maximum) values for measured variables and frequency and percentage (%) for categorical data.

## Results

A total of 308 out of 329 AS patients were included in this study. Twenty-one patients were not included in the study because they could not clearly remember their smoking history. The mean age of AS patients participating in the study was 42.4 ± 11.0 years, with 158 (51.3%) being male. The patients' mean age of disease onset was 35.8 ± 11.0 years. The median disease duration of the patients was 4.4 (0.5-22.5) years. The number of HLA-B27 in 161 patients was found to be 123 (76.3%). The number of smokers was 108 (35.1%), the number of former smokers was 102 (33.1%), the number of non-smokers was 37 (12.0%) and 61 (19.8%) did not provide information about smoking. A total of 247 patients (80.2%) stated that the rheumatologist questioned them about smoking and informed the rheumatologist about this issue. The smoking duration of current smokers was 13.7 ± 9.2 years, and the smoking duration of former smokers was 23.7 ± 11.6 years. The demographic and clinical data are shown in Table 1.

**Table 1:** Baseline characteristics of AS patients (N = 308) Data are presented as mean ± standard deviation, median (minimum–maximum), or n (%) NSAID: Non-steroidal anti-inflammatory drug, DMARDs: Disease-modifying antirheumatic drugs, BMI: body mass index, AS: Ankylosing spondylitis, boDMARD: Biologic originator disease-modifying antirheumatic drugs

Parameter	Value
Age, years mean ± SD	42.4 ± 11.0
Males, n (%)	158 (51.3)
BMI (kg/m^2^), mean ± SD	27.1 ± 4.0
Comorbidity, n (%)	107 (34.7)
Disease onset age, years mean ± SD	35.8 ± 11.0
Disease duration, years	4.4 (0.5-22.5)
Marital status, n (%)	
Married	250 (81.2)
Unmarried	58 (18.8)
Education, n (%)	
Uneducated	28 (9.1)
Primary school	42 (13.6)
High school	108 (35.1)
University	132 (42.9)
HLA-B27 positivity, n (%)	123 (76.3)
Treatment used for AS, n (%)	
NSAIDs/DMARDs	132 (42.9)
boDMARDs	176 (57.1)
Alcohol, n (%)	44 (14.3)
Smoking status, n (%)	
Current-smokers	108 (35.1)
Former-smokers	102 (33.1)
Non-smokers	37 (12.0)
Not asked	61 (19.8)
Per/day smoking	12 (5-45)
Pocket/years	10 (0.5-40.0)

In further analysis, the reasons for quitting smoking of 102 AS patients were evaluated, the leading causes for quitting smoking were the rheumatologist's recommendation in 39 (38.3%), and concerns about the disease itself in 29 (28.4%) patients (Table 2). The most commonly used methods for quitting smoking were 40 (39.2%) medications for smoking cessation and 40 (39.2%) herbal supplements. The medical treatment given to patients who want to quit smoking is provided by health professionals appointed by the Ministry of Health. The second most frequently used method of smoking cessation was the patient's own request (Table 2).

**Table 2:** Factors leading to quit smoking and smoking cessation methods (N = 102) * Personal concerns about the disease itself

Factors	n (%)
Rheumatologist's advice	39 (38.3)
Ankylosing spondylitis*	29 (28.4)
Patient's request	23 (22.5)
Other diseases	11 (10.8)
Methods	
Medication for smoking cessation	40 (39.2)
Herbal supplement	40 (39.2)
Psychotherapy	18 (17.6)
Acupuncture, sport	4 (3.9)

## Discussion

This study shows that most of the AS patients were advised to quit smoking by the rheumatologist, and it was determined that the most influential factor in quitting smoking for these patients was the recommendation of the rheumatologist. In addition, the study shows that the most commonly used methods of smoking cessation in AS patients were found to be medication for smoking cessation and herbal supplements.

Smoking is a serious public health problem in Turkey and one of the most important environmental risk factors for rheumatologic diseases [9]. Many studies have been conducted to investigate the impact of smoking on AS disease. Smoking not only contributes to the progression of AS but is also thought to play a role in its development [9-12]. In the study, it was observed that Turkish and Egyptian smokers diagnosed with AS had higher BASDAI scores than non-smokers. It showed that longer smoking duration in smokers with AS was associated with a higher risk of disease activity, and disease activity increased more as the years of smoking increased [13].

The research conducted in six European countries on doctors' questions about smoking and directing patients to quit smoking showed that only 37.7% of patients received a brief recommendation from their doctor. Besides, in that study, 25% of current smokers reported that they did not get advice to quit smoking from their doctor even when questioned about smoking [14]. In the online study conducted by inflammatory bowel disease Partners in the USA between 2011 and 2014, 453 inflammatory bowel disease patients who defined themselves as current smokers were asked whether they had received medical advice to quit smoking retrospectively. Overall, about 77% of smokers reported that they had gotten medical advice to quit smoking [15].

In another study, a prospective cohort analysis was performed with data obtained from the Danish smoking cessation database, which included patients with rheumatoid arthritis and osteoarthritis. Of the 24,652 patients included in this study, 65% were advised to quit smoking by health personnel (doctor, nurse, nurse assistant, midwife, etc.) [16]. In a study by Naranjo et al. [17] involving 28 rheumatology departments, 64% of doctors recommended quitting smoking. In a study involving 395 rheumatologists from 16 European countries, it was determined that 64.5% of the patients received smoking cessation advice. Fewer than 25% of departments had a specific smoking cessation consensus protocol or written recommendation to quit smoking. Rheumatologists in high gross domestic product countries and centers with early arthritis clinics were more likely to advise most smokers [18]. In our study, the questioning of the smoking status of the patients by the rheumatologist was higher than in other studies. Moreover, 38.3% of these patients quit smoking due to the recommendation of a rheumatologist, which emphasizes the importance of the role of the attending physician. Although the contribution of the rheumatologist is quite efficient, there are patients who were not questioned about their smoking status, constituting almost 1/5 of the total smokers, and this is the other side of the medallion. It can be speculated that, if all of the patients were asked if they smoked, the rate of quitting could rise to nearly 50%. As a result, rheumatologists should be aware of both the negative impact of cigarette smoking on the progression of AS disease and their own influence on patients' motivation to quit.

There are different and somewhat effective modalities used to quit smoking, including psychotherapy, pharmacotherapy, acupuncture, etc. In a review of 53 studies, pharmacotherapy (nicotine replacement therapy, bupropion, nortriptyline, and varenicline) was found to be an important smoking cessation method when compared to control groups [19]. In a study that included 120 cigarette-dependent patients, acupuncture was found to be an effective method for smoking cessation. In addition, age, education level, and nicotine metabolite ratio were effective factors in quitting smoking in the intervention with acupuncture [20]. In a network meta-analysis, pharmacotherapy provided a significant success rate in smoking cessation [21]. Eighty one percent of 324 patients diagnosed with chronic obstructive pulmonary disease quit smoking within one year with pharmacotherapy (nicotine patch, nicotine gum, bupropion, varenicline tartrate, bupropion, and nicotine patch in combination) [22]. In our study, 39.2% of AS patients quit smoking with medical treatment and 39.2% with herbal treatment methods. This study is important in terms of showing that nonpharmacological methods were used as commonly as pharmacological agents in patients with AS.

There are some limitations of the study. This is a single-center study, and the number of patients is limited. Disease activity criteria of the patients were not evaluated. On the other hand, since it is a descriptive study, patient's inability to remember may affect the results. Therefore, these issues should be taken into account when evaluating the results of the study.

## Conclusions

It has been determined that smoking, which is an important and modifiable factor in the prognosis of the disease, is not evaluated in a significant portion of the patients. On the other hand, it was observed that the factor that had the greatest effect on those who quit smoking was the rheumatologist’s recommendation. Regardless of the reason for admission, every contact with the patient should be considered as an opportunity to combat the health risks posed by smoking. In this sense, it is thought that rheumatologists' questioning of smoking in AS patients and suggesting smoking cessation will contribute to disease control and long-term medical outcomes.
